# A measure for intrinsic information

**DOI:** 10.1038/s41598-020-75943-4

**Published:** 2020-11-02

**Authors:** Leonardo S. Barbosa, William Marshall, Sabrina Streipert, Larissa Albantakis, Giulio Tononi

**Affiliations:** 1 Department of Psychiatry, University of Wisconsin-Madison, Madison, WI 53719 USA; 2 Department of Mathematics and Statistics, Brock University, St. Catharines, ON L2S 3A1 Canada; 3 Department of Mathematics and Statistics, McMaster University, Hamilton, ON L8S4K1 Canada

**Keywords:** Information technology, Neuroscience

## Abstract

We introduce an information measure that reflects the intrinsic perspective of a receiver or sender of a single symbol, who has no access to the communication channel and its source or target. The measure satisfies three desired properties—causality, specificity, intrinsicality—and is shown to be unique. Causality means that symbols must be transmitted with probability greater than chance. Specificity means that information must be transmitted by an individual symbol. Intrinsicality means that a symbol must be taken as such and cannot be decomposed into signal and noise. It follows that the intrinsic information carried by a specific symbol increases if the repertoire of symbols increases without noise (expansion) and decreases if it does so without signal (dilution). An optimal balance between expansion and dilution is relevant for systems whose elements must assess their inputs and outputs from the intrinsic perspective, such as neurons in a network.

## Introduction

**Figure 1:**
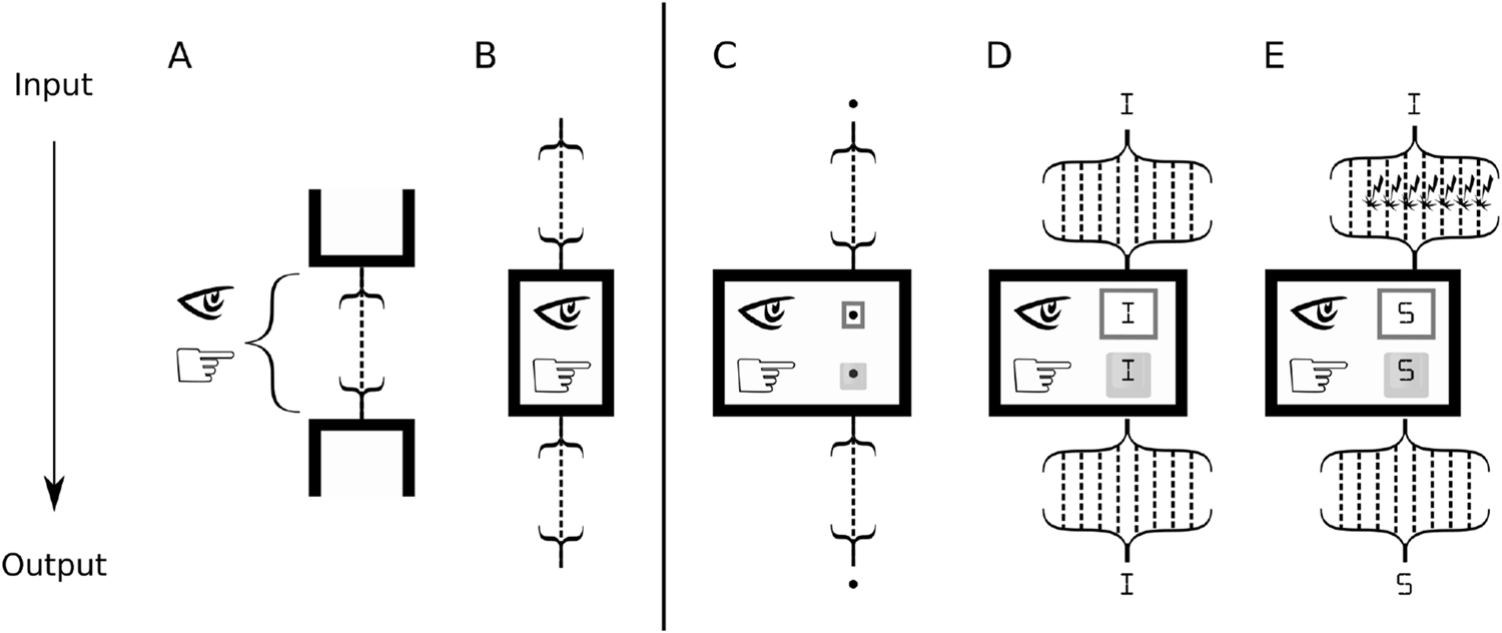
Information from the intrinsic perspective. Different channels constituted of wires carrying a physical signal with two states (for example, high or low voltage); the brackets pointing up represents the encoding of the symbols into the physical signal(s) and the brackets pointing down represent the decoding. (**A**) Extrinsic perspective: encoders and decoders can be designed to find the alphabet that maximizes information transmission (channel capacity), and this alphabet is shared by both sender and received. (**B**) Intrinsic perspective: has fixed encoders and decoders for sending and receiving information. The remaining panels depict three enclosures where your only interface with the environment is an input channel (top) connected to a screen and an output channel (bottom) connected to a keyboard. (**C**) a channel with one noiseless wire, conveying one bit that is encoded (decoded) into a symbol (a dot or a dash); (**D**, **E**) channels with eight wires each and an encoder (decoder) that converts the eight bits (a byte) into one out of 256 characters (or vice-versa). In (**D**), all eight wires are noiseless. In (**E**), the first wire is noiseless while the other seven wires are fully noisy (indicated by the lightning signs) so they rarely convey the original signal. In all cases the keyboard is connected to noiseless output wires (bottom) and has as many keys as possible input symbols. How should you rank the three enclosures with respect to the information you could relay?

Information measures were originally designed to optimize artificial communication channels (Fig. 1A), where a sender and a receiver can freely agree upon a code, implemented by a channel designer, in order to optimize the amount of information transmitted between them^1^. More recently, these measures have been extended to natural systems^2,3^—for example, sensory and motor pathways in the brain^4^. Here, we consider such systems from a different point of view and propose a measure that captures information from the intrinsic perspective (Fig. 1B) of elements *within* a system—for example, neurons in the cerebral cortex.

To get an intuitive sense of how to measure information from the intrinsic perspective, consider the following thought experiment. You will be sealed inside an enclosure where your only interface with the environment is a screen for receiving input from a source and a keyboard for sending output to a target. Once inside the enclosure, your task will be to relay as much information as possible from source to target. The enclosure has an input and an output channel, constituted of wires carrying a physical signal with two states (for example, high or low voltage). For each combination of signals arriving through the input wires (input state) the enclosure has a fixed decoder which converts the signal into one specific symbol displayed by a screen. The keyboard has a matching set of keys with symbols converted into signal by a fixed encoder and transmitted through the output wires (output state). The enclosure, with its wires, encoder and decoder, screen and keyboard, defines a physical channel to be used for communication, analogous to a neuron with its axon, dendrites and firing mechanism. Like neurons in a network, no further communication or agreement is permitted between a sender and the person inside the enclosure. For example, the sequence of messages cannot be adapted to form an error-correcting code for the channel.

Suppose you can choose from three enclosures. The first enclosure (Fig. 1C) has an input channel with one wire, so the screen can display one of two input symbols (say, a dot or a dash). The wire in this channel is noiseless, meaning that whatever you see on the screen is the symbol sent by the source. The second enclosure (Fig. 1D) has an input channel with eight wires, so the screen can display any of 256 possible input symbols (say, ASCII characters). Again, all the wires are noiseless and whatever you see on the screen is the symbol sent by the source. The third enclosure (Fig. 1E) also has an input channel with eight wires, so the screen also displays any of the 256 possible input symbols. However, in this case the seven additional wires carry no signal (only noise), and the odds of seeing the symbol sent by the source are 1:127. In all cases, the output wires are noiseless. Given that you will be sealed inside the enclosure without access to the outside (to input and output wires, source or target), which enclosure should you choose to relay as much information as possible?

The second enclosure is clearly best, as it has a larger repertoire of possible input symbols than the first enclosure and is also noiseless, unlike the third enclosure. But suppose that the second enclosure is already taken and you have to choose between the first and third enclosure. Standard measures of information imply that, from the extrinsic perspective of a channel designer, the input channels of the first and third enclosures are equivalent, since both can potentially transmit one bit per symbol when using proper error correction. However, things are different from your intrinsic perspective within the enclosure, where you only have access to the input on the screen and the output on the keyboard. From your perspective, the input on the screen has to be taken as such and cannot be decomposed into signal and noise (the lack of extrinsic access to the channel in Fig. 1 is indicated by brackets pointing down, which represent the decoding of the signals carried by the wires into a single symbol on the screen). From your perspective, while in the first enclosure the input channel transmits the correct symbol on every transmission, in the third enclosure the information from the noiseless wire is irreparably contaminated by the noise in the other wires. On average, the input channel in the third enclosure transmits one correct byte every 128 transmissions, while the first enclosure transmits one correct byte every eight transmissions.

In what follows, we develop a measure of intrinsic information (information from the intrinsic perspective), which captures the intuition from the above example. We show that this can be done by a measure that satisfies three properties.

- *Causality* The measure should differ from zero only if the the symbols are transmitted with probability greater than chance.
- *Specificity* The measure should reflect how much information is transmitted by an individual symbol.
- *Intrinsicality* The measure should increase if the repertoire of symbols increases without noise (expansion) and decrease if it does so without signal (dilution).

In “Theory” we derive a unique measure of intrinsic information, called the *intrinsic difference* (ID), by mathematically formulating the causality, specificity, and intrinsicality properties. Then, in “Results and discussion” we explore the behavior of the ID measure.

## Theory

In this section we introduce the measure of the intrinsic information, relying on the previous example for motivation. The formal mathematical definition of the measure is presented in the “Supplementary Materials”. Let *X* be the symbol selected by the sender and *Y* be the symbol displayed on the screen in the enclosure. Both *X* and *Y* are discrete random variables with support $\Omega$. We define the desired properties for a measure of intrinsic information in terms of the conditional probability distribution for the symbol $x_\alpha \in \Omega$ selected by the sender given that symbol $y_\beta \in \Omega$ was observed on the screen, $P^n = [p_1, \ldots , p_n]$ where

$$\begin{aligned} p_\alpha = P(X = x_\alpha | Y = y_\beta ), \end{aligned}$$

and the marginal distribution for the symbol selected by the sender $Q^n = [q_1, \ldots , q_n]$, where

$$\begin{aligned} q_\alpha = P(X = x_\alpha ). \end{aligned}$$

The marginal distribution $Q^n$ represents a lack of causal connection between the source and the screen, i.e., the distribution that would arise if all wires were transmitting noise. For the enclosure example we have in mind that $q_\alpha = 1/n$, but this does not need to be the case and may depend on the specific details of the channel (see “Intrinsic information among network elements”). Moreover, let $V^n = [1, 0, \ldots , 0]$ and $U^n = [1/n, \ldots , 1/n]$; then a channel is called noiseless if $P^n = V^n \ne Q^n$, or fully noisy if $P^n = U^n = Q^n$. We now provide formal definitions for three properties that must be satisfied by a measure of intrinsic information. We only consider the receiver (input) side of the sealed enclosure and the source of the signal, but the same properties can be equally defined with respect to the sender (output) side and the target.

- *Causality* The measure $D(P^n,Q^n)$ should only be zero when the source has no causal connection to the symbol on the screen. In other words, when the probability of *any* symbol possibly transmitted by the source given the symbol observed on the screen is equal to chance level. More formally,
  $$\begin{aligned} D(P^n, Q^n) = 0 \iff P^n \equiv Q^n. \end{aligned}$$
- *Specificity* The source transmits one specific symbol at a time. Accordingly, the measure $D(P^n,Q^n)$ should reflect the information contained in *one specific symbol* as opposed to some collective property, such as the average over all possible symbols. We assume that the source and the receiver want to maximize the information received from each transmission, and thus the measure should reflect the symbol for which intrinsic information is maximal. Let $f(p_\alpha ,q_\alpha )$ be the function representing how much intrinsic information is gained about each symbol $x_\alpha$ possibly chosen by the source, given the symbol $y_\beta$ observed on the screen. We then define
  $$\begin{aligned} D(P^n, Q^n) = \max _{\alpha \in \{1, \ldots , n\}} \{f(p_\alpha , q_\alpha )\}. \end{aligned}$$
- *Intrinsicality* From the intrinsic perspective, one has no access to the communication channel and its source or target; hence each symbol must be taken as such and cannot be decomposed into signal and noise. Accordingly, intrinsic information should increase if the channel is extended by adding noiseless signal, but should decrease if it is extended by noise. By extension we mean that some original channel $P^n_1$ is extended by another independent channel $P^m_2$ forming a new channel $P^n_1 * P^m_2$ (where $*$ is the Kronecker product) with a larger repertoire of symbols. If a noiseless channel $P^n_1 = V^n_1$ with chance level $Q_1^n$ is extended by another noiseless channel $P^m_2 = V^m_2$ (independent of $V^n_1$) with chance level $Q_2^m$ (independent of $Q_1^n$), then the resulting channel $V^n_1 * V^m_2$ with chance level $Q_1^n * Q_2^m$ has a larger symbol repertoire and still transmits a noiseless signal from the source. Thus, extending a channel in a noiseless way increases (*expands*) intrinsic information. More formally, in this case the measure should be additive, meaning
  $$\begin{aligned} D(V^n_1 * V^m_2, Q^n_1 * Q^m_2) = D(V_1^n, Q_1^n) + D(V_2^m, Q_2^m). \end{aligned}$$
  Conversely, if a channel $P^n$ with chance distribution $Q^n$ is extended by a fully noisy channel $U^m$ with chance distribution also $U^m$, the resulting channel $P^n * U^m$ with chance level $Q^n * U^m$ has a larger repertoire but no new signal. In this case, the new channel is *m* times less likely to correctly transmit the original signal sent by the source, and the information should be reduced by a factor *m*. In other words, extending a channel in a fully noisy way decreases (*dilutes*) intrinsic information. More formally, in this case the measure should be sub-additive, meaning
  $$\begin{aligned} D(P^n * U^m, Q^n * U^m) = \frac{D(P^n, Q^n) + D(U^m, U^m)}{m}. \end{aligned}$$

Our main result is the existence of a unique function (up to a multiplicative constant $k > 0$)

1 $$\begin{aligned} D(P^n,Q^n) = k \max _\alpha \left\{ p_\alpha \log \left( \frac{p_\alpha }{q_\alpha } \right) \right\} , \end{aligned}$$

that satisfies all three properties. We set $k=1$ without loss of generality and call this function *intrinsic difference* (ID). The full statement of the theorem and its proof are presented in the “Supplementary Materials”. Although our properties only impose constraints on the behaviour of the ID measure for specific distributions $P^n = P(X|Y=y_\beta )$ and $Q^n = P(X)$, by identifying a unique functional form we can extrapolate these properties across the entire domain of probability distributions, as shown below.

The form of the ID measure is reminiscent of classical information measures, such as the Kullbeck–Leibler (KL) divergence

$$\begin{aligned} \text{KL}(P^n,Q^n) = \sum _{\alpha =1}^n p_\alpha \log \left( \frac{p_\alpha }{q_\alpha }\right) . \end{aligned}$$

The KL divergence can be interpreted as a weighted average of the difference in Hartley information $\log (p_\alpha / q_\alpha )$ for each state $\alpha$, weighted by its probability $p_\alpha$. Similarly, the ID measure can be interpreted as the information density associated with the optimal state $\alpha$, where the information $\log (p_\alpha / q_\alpha )$ associated with that state (informativeness) is multiplied by the probability density at that state $p_\alpha$ (selectivity). Drawing an analogy between information and mass, while the KL measure computes the total mass of the system, the ID measure finds the point with the highest density anywhere in the system.

## Results and discussion

We introduced a novel information measure, which we called the intrinsic difference (ID; Eq. 1) because it captures the difference between $P^n$ and $Q^n$ from the intrinsic perspective. The measure satisfies three basic properties, *causality*, *specificity*, and *intrinsicality*, and is unique. Note that these properties are different from the ones commonly required in classical information theory (see “Comparison to other information measures” for a detailed discussion of other measures). We now investigate the behavior of this measure when applied to the example presented in Fig. 1 and to other similar situations. We then consider a scenario in which a neuron chooses its optimal fan-out according to the ID. Finally, we discuss the behavior and form of the ID in the context of current information measures.

### Intrinsic information

The example in Fig. 1 posed a choice between three input channels with different numbers of wires. Let $r_i$ be the probability of each wire correctly transmitting the bit sent by the source in a given channel, where $i \in \{1,\ldots ,N\}$ and N is the number of wires in the channel. The first channel is a noiseless one-wire channel (bit-size) which transmits the original signal with probability one, that is $r_1 = 1$ and the conditional distribution is $P^2 = V^2$. The second channel is a noiseless eight-wire channel (byte-size) which has 7 additional wires that also transmit the original signal with probability one, that is $r_i = 1, i \in \{1,\ldots , 8\}$ and $P^{2^8} = V^{2^8}$. Finally the third channel is a noisy byte-size channel which also has one noiseless wire but seven fully noisy wires that transmit the original signal with probability half, that is $r_1 = 1$ and $r_i = \frac{1}{2}, i \in \{2, \ldots , 8\}$. We assume the noise to be independent and identically distributed (iid) in all wires (but see “Supplementary Materials Figure S1” for an example of correlated noise). The conditional distribution for the third channel is $P^{2^8} = [p_1, \ldots , p_{2^8}]$ with

$$\begin{aligned} p_\alpha = {\left\{ \begin{array}{ll} 1/2^7 &{}\quad \text {if } 1 \le \alpha \le 2^7 \\ 0 &{}\quad \text { otherwise.} \end{array}\right. } \end{aligned}$$

**Figure 2:**
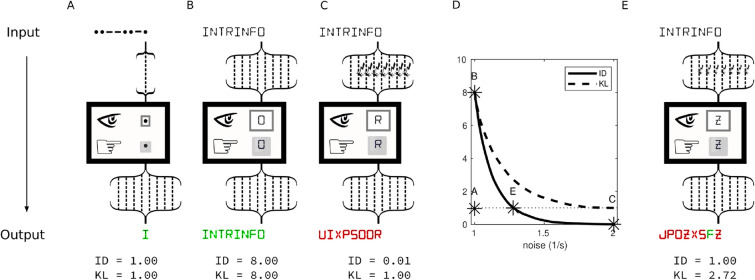
Intrinsic information for different levels of noise. To facilitate comparison between input channels and to provide a read-out of the enclosure, all output channels are eight-bit noiseless wires that reproduce exactly what was sent. For bit-sized input channels, eight key presses are accumulated in the encoder (bracket pointing up) to reproduce one output character every eight bits, representing a common read-out at the target (bracket pointing down). The colors represent whether the output character is correct (green) or incorrect (red). (**A**) Similar to Fig. 1C, a one-wire input channel that is noiseless, meaning it transmits the original signal with probability one (noiseless bit-channel). (**B**) Similar to Fig. 1D, an eight-wire input channel with seven additional wires that are also noiseless and transmit the original signal with probability one (noiseless byte-channel). (**C**) Similar to Fig. 1E, an input channel with one noiseless wire and seven fully noisy wires that transmit the original signal with probability half (noisy byte-channel). Both the intrinsic difference measure (ID) and the KL measures attribute the same amount of information to the noiseless bit-channel and the noiseless byte-channel (1 and 8, respectively). However, the KL measure attributes 1 bit to the noisy byte-channel, whereas the ID measure attributes to it close to 0 *ibits*. This is because the ID reflects the amount of information available from the perspective of the receiver in the sealed enclosure (intrinsic information), whereas KL reflects the information available from the extrinsic perspective of a channel designer, which could be extracted using error correcting codes. (**D**) A byte-channel with additional noisy (iid) wires that transmit the original signal with decreasing probability $r_i = s, i \in \{2, \ldots , 8\}$ (or increasing levels of noise $\frac{1}{s}$), from $s=1$ (noiseless) to $s=\frac{1}{2}$ (fully noisy). Due to the additivity property, the KL measure (dashed line) will always attribute at least 1 bit of information to the noisy-byte channel. By contrast, ID starts from nearly 8 *ibits* and decreases to nearly 0 *ibits*. (**E**) Due to the smoothness of ID, there is a level of noise ($s \approx 0.78$) for which a byte-channel conveys 1 *ibit*. On a typical run with eight transmissions such a byte-channel outputs one correct byte-size character, similar to the noiseless bit-channel.

In all cases, we assume that $Q^n = U^n$. Note that for this choice of $Q^n$, the value of $D(P^n, Q^n)$ is the same for each possible symbol that could appear on the screen (e.g., for each $y_\beta \in \Omega$), but this is not generally the case (see “Intrinsic information among network elements”). Moreover, note that the choice of one noiseless wire and seven additional noisy wires is arbitrary and only serves to facilitate comparisons with our motivating example. Other arrangements, for example two noiseless and six noisy wires, do not change the qualitative behavior (see “Supplementary Materials Figure S2”). To facilitate intuitive comparisons between these three input channels, we take advantage of an output channel that outputs one byte-size character for every eight bits of input signal it accumulates (Fig. 2), representing a common target read-out.

The KL and ID measures assign 1 bit and 1 intrinsic bit (*ibit; since the quantities returned by the KL and ID functions are not commensurable, we define the new unit intrinsic bit, or ibit for short, to name the quantity returned by the ID function*) to the noiseless bit-size channel, respectively (Fig. 2A). They also assign 8 bits and 8 *ibits* to the noiseless byte-size channel (Fig. 2B). However, while the KL measure assigns 1 bit to the noisy byte-size channel, the ID measure assigns close to zero *ibits* (Fig. 2C). This result agrees with the intuition described in “Introduction”.

### Intrinsic information and noise level

Figure 2D shows how KL and ID change when one wire of a byte-size channel is noiseless ($r_1 = 1$) and the other seven wires have noise (iid) varying from noiseless to fully noisy, that is, for $s \in \left[ 1, \frac{1}{2}\right]$,

$$\begin{aligned} r_i = s, \quad i \in [2, \ldots , 8]. \end{aligned}$$

The conditional distribution for such a channel is $P^{2^8} = [p_1, \ldots , p_{2^8}]$ where for

$$\begin{aligned} p_\alpha = {\left\{ \begin{array}{ll} s^7 &{} \text {if } 1 \le \alpha \le 2^7 \\ 0 &{} \text { otherwise.} \end{array}\right. } \end{aligned}$$

Chance level is $Q^{2^8} = U^{2^8}$. Because the KL measure is additive, its value for all intermediate levels of noise is greater than or equal to the value of the noiseless bit-size channel, regardless of how often the receiver sees the correct symbol. The KL measure reflects the possibility of recovering the noiseless bit using error correcting codes implemented through an agreement between the source and the receiver. By contrast, because the ID measure is sensitive to added signal (expansion) and added noise (dilution), its value at intermediate levels of noise can be lower or higher than that of a noiseless bit-size channel. Moreover, because the measure is smooth, there must be an intermediate noise level at which the byte-size channel is equivalent to the noiseless bit-size channel. A noisy byte-size channel with $s \approx 0.78$ yields 1 *ibit* of intrinsic information (Fig. 2E), and similar to the noiseless bit-size channel, on a typical run with eight transmissions its output will display one correct byte-size character.

### Intrinsic information and channel size

**Figure 3:**
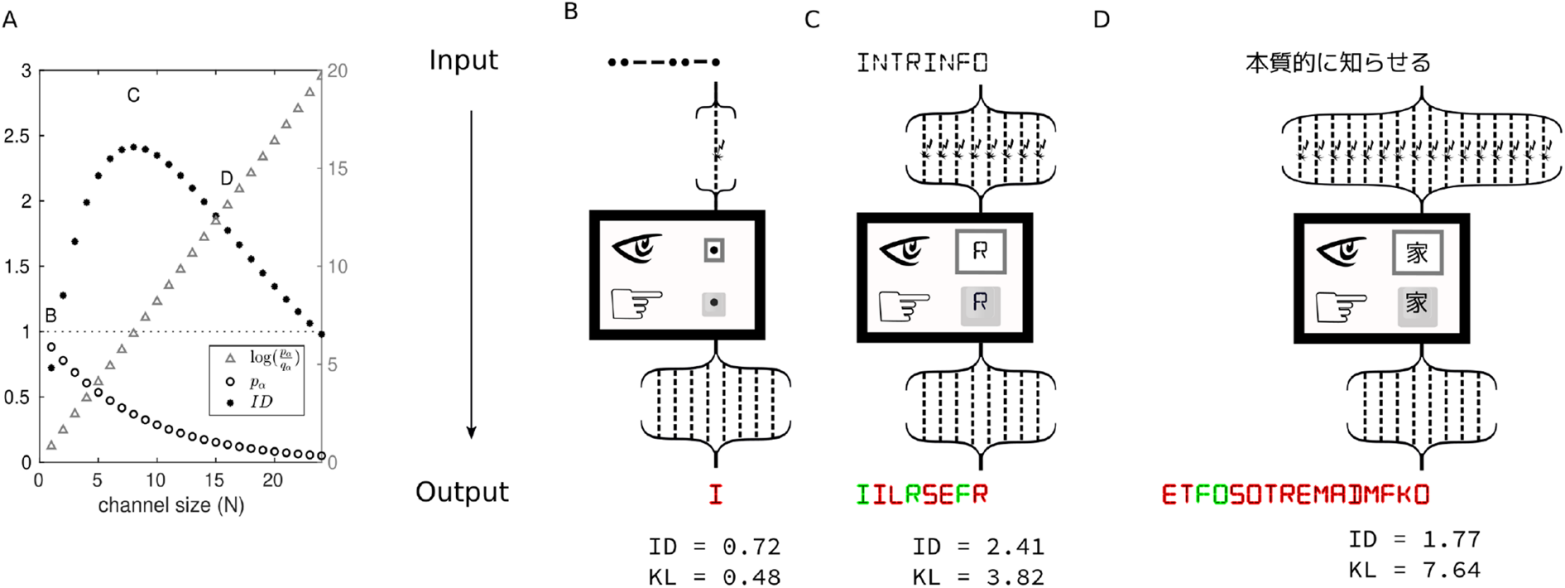
Intrinsic information for different channel sizes. As before, all output channels are eight-bit noiseless wires that reproduce exactly what was sent (common read-out), and the colors represent whether the output character is correct or not. (**A**) A balance between expansion and dilution can be seen for an increasing number *N* of wires, given a fixed level of noise per wire (the probability of receiving the correct signal in each wire is $r = 0.88$). As the informativeness $\log \left( \frac{p_\alpha }{q_\alpha }\right) = N \log \left( \frac{r}{2}\right)$ grows linearly and the selectivity $p_\alpha = r^N$ decreases exponentially, their product ID (black dots) peaks at $N = 8$ wires. (**B**) On a typical run with 8 transmissions, a channel with too few input wires (one) does not output any correct byte-size character; (**C**) a channel with an optimal number of wires (eight) maximizes ID and outputs three correct ASCII characters; (**D**) a channel with too many input wires (16), could transmit thousands of different Kanji characters (for instance using unicode), but ID is lower and the enclosure outputs only two correct ASCII characters after eight transmissions.

Next, we consider a scenario where channels have different sizes *N* and the probability of each noisy wire (iid) transmitting the bit selected by the source is fixed at $r_i = s$ (say, $s = 0.88$; Fig. 3A). The conditional distribution for this channel is $P^{2^8} = [p_1, \ldots , p_{2^8}]$ where

$$\begin{aligned} p_\alpha = s^{a_\alpha }(1-s)^{N - a_\alpha }, \end{aligned}$$

and $a_\alpha$ is the number of bits that symbol $y_\beta$ on the screen has in common with the symbol $x_\alpha$ selected by the source. Chance level is $Q^{2^8} = U^{2^8}$. For $s \ge 0.5$, the maximal value occurs for the symbol selected by the source and $\max (p_\alpha ) = s^N$. In this situation a one-wire, bit-size channel ($N=1$; Fig. 3B) conveys approximately 0.72 *ibits*, and on a typical run of eight transmissions the output character will not be correct. A 16-wire, two-byte channel employing unicode symbols, which could for instance transmit thousands of different symbols from the Kanji alphabet, conveys 1.77 *ibits* (Fig. 3C). On a typical run with eight transmissions, the output will contain two correct byte-size characters. The maximum amount of intrinsic information (2.41 *ibits*; Fig. 3D) is conveyed by an eight-wire, byte-size channel, where the output on typical run will contain three correct byte-size characters. Notice that the ID measure reveals a trade-off between the maximum amount of information conveyed by a single symbol (informativeness $\log (p_\alpha /q_\alpha )$; Fig. 3A) and how often such symbol is correct (selectivity $p_\alpha$; Fig. 3A). This trade-off is resolved optimally when the expected amount of information correctly received is maximized from the perspective of the receiver.

Due to additivity, in this example the KL measure will monotonically increase with *N*. Using maximum KL to select the channel size will always pick the one with largest *N*, which makes sense when an optimal encoding/decoding is available to recover any information that is corrupted by the noise. Although such an encoding/decoding is guaranteed to exist^1^, it is not always possible to implement it in an algorithm within the system. When the optimal encoding is not available, a common solution involves specifying the maximum acceptable error rate for a channel (one-shot transmission^5^). In this situation, which is common in real-time communications^6^, a channel is selected by maximizing the KL measure subject to the constraint that the error rate of the channel is below the maximum acceptable error rate (see “Supplementary Materials Section 1.3”).

Here we demonstrated an alternative solution based on maximizing intrinsic information that does not require a subjective constraint on the maximum error rate of the channel. In this example, the optimal channel identified by the ID measure (Fig. 3C) has an error rate which maximizes the number of symbols correctly received by the receiver. Crucially, from the intrinsic perspective, the bits received are only informative if they contribute to transmitting the correct symbols selected by the source. In this case, the receiver must use the symbols as is, without extra processing to extract the correct bits (selectivity). At the same time, the larger the alphabet originating the symbol sent by the source, the more informative the symbol can be (informativeness). By using the unique measure that satisfies the three properties for intrinsic information, our solution arrives at a specific, principled error rate for the optimal channel, which may be useful in several applications.

### Intrinsic information among network elements

**Figure 4:**
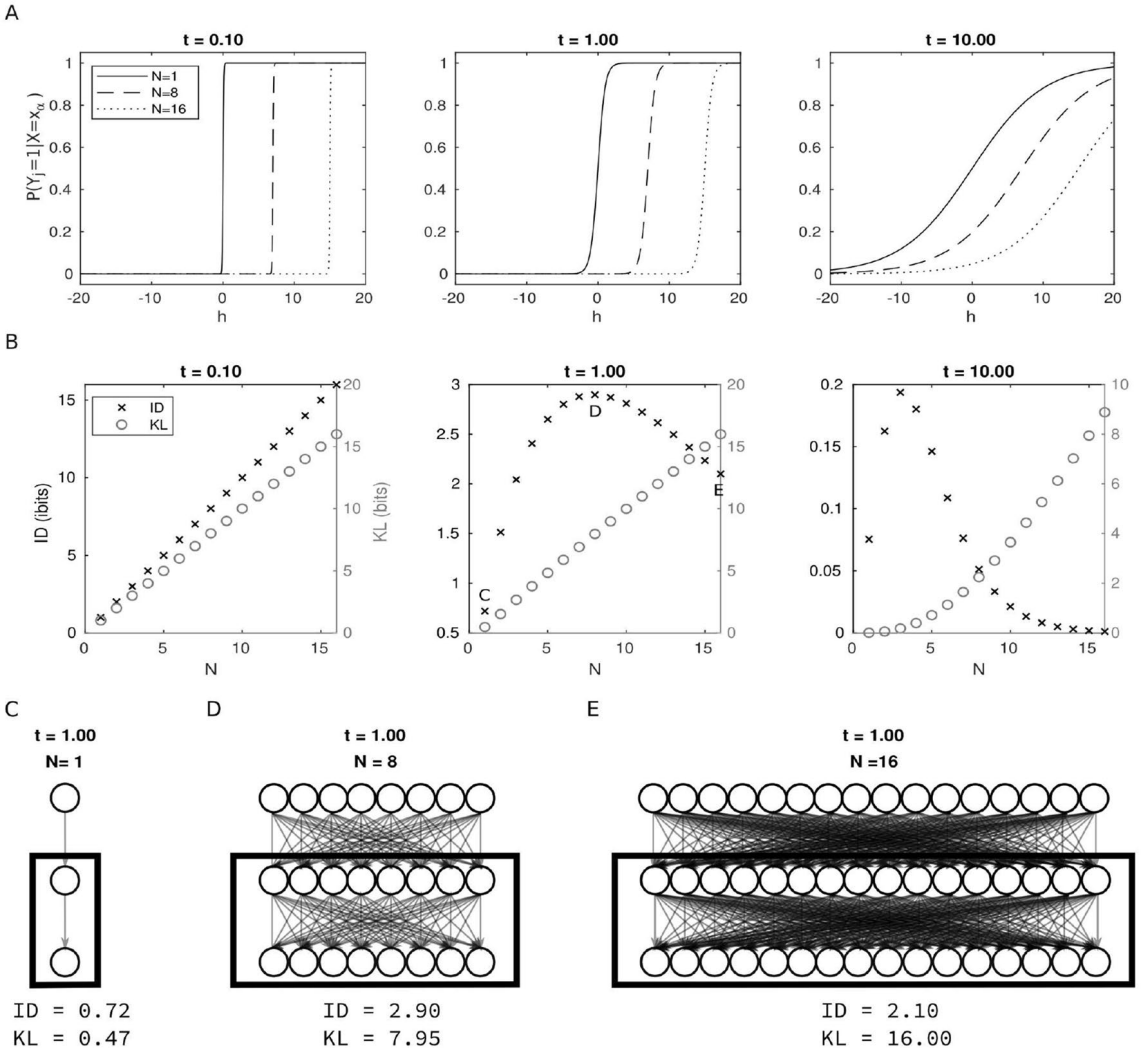
Information from the intrinsic perspective of elements in a system. (**A**) Probability of firing of one simplified neuron, given the input states for three different input sizes *N* and three different levels of noise (or value of parameter *t*). (**B**) Information measured by the intrinsic difference (ID) and KL divergence (y-axis) for groups of simplified neurons with varying numbers of outputs *N* (x-axis). The number of neurons in the group is always the same as the number of neurons in the output. Each panel shows the behavior of ID and KL for different levels of noise. Notice that each panel has two y-axis, one for each measure, since they are not commensurable (*bits* and *ibits*) and the goal is not to compare their absolute values, but their qualitative behavior: While KL always reaches its maximum values when the neurons are connected to all available outputs, ID shows a peak that changes with the level of noise. (**C**) For the noise level $t = 1$, neurons with one output convey 0.72 *ibits*; (**D**) the maximum of ID is reached by neurons with 8 outputs (2.90 *ibits*), again showing a balance between expansion and dilution; (**E**) neurons with 16 outputs have lower values of ID (2.10 *ibits*) than those with eight outputs.

Consider now the situation in which information is assessed, rather than from the intrinsic perspective of a human in an enclosure, from the intrinsic perspective of elements in a complex physical system, such as neurons in a brain. We consider a network of simplified neurons, where each neuron has *N* inputs, *N* outputs, and the same firing mechanism. We model the probability of a single neuron $Y_j$ firing ($Y_j = 1$) as opposed to being silent ($Y_j = -1$) using a sigmoidal activation function^7, 8^

$$\begin{aligned} P(Y_j = 1|X = x_\alpha ) = \frac{1}{1 + \exp \left[ -2t^{-1}(h(x_\alpha ) + b)\right] }, \end{aligned}$$

where $X = \{X_1, X_2, \ldots , X_N\}$ is the set of *N* inputs to neuron $Y_j$, $x_\alpha \in \{-1,1\}^N = \Omega$ is one specific input state, $h(x_\alpha ) = \sum _{i=1}^N x_{\alpha ,i}$ is the contribution of the input neurons towards firing ($Y_j = 1$), *t* is the slope of the sigmoid, and *b* is a bias towards firing more or less frequently (Fig. 4A). We select the bias $b = 1 - N$, which represents a biological limit in the probability of firing when all inputs are active, given by the slope *t*. This choice simplifies the interpretation of the results, since the probability of firing (noise level) is only manipulated through the slope *t*, allowing independent manipulation of the number of output neurons *N* (size of the repertoire).

While in “Intrinsic information and noise level” and “Intrinsic information and channel size” we focused on the receiver’s perspective (information $Y_j$ receives from *X*), we now focus on the sender’s perspective (information *X* sends to $Y_j$). More specifically, we can study the intrinsic information transmitted from a set of neurons in a particular state $x_\alpha = 1^N= \{1,\ldots ,1\}$ to a different output set $Y = \{Y_1, Y_2, \ldots Y_N\}$ through the conditional probability

$$\begin{aligned} P^n = P(Y|X=1^N) = \bigotimes _{j=1}^N P(Y_j|X=1^N), \end{aligned}$$

where $n = 2^N$, $P(Y_j|X=1^N)$ is the conditional distribution of the *j*th output and $\bigotimes$ indicates the Kronecker product between all such distributions. We take the chance level distribution to be

$$\begin{aligned} Q^n = P(Y) = \sum _{x_\alpha \in \{-1,1\}^N} P(Y|X=x_\alpha ) P(X=x_\alpha ), \end{aligned}$$

where $P(X = x_\alpha ) = 1/n$ is the uniform distribution. We assume iid noise, leading to output neurons that are conditionally independent given the state of the input neurons ($P^n$); however, they are not independent in the joint (unconditional) distribution ($Q^n$), this is because the output neurons have common inputs and thus carry redundant information.

Figure 4B shows the results for three different levels of noise. The ID measure peaks at different values of *N* depending on the noise level—the higher the level of noise, the lower the number of outputs for peak ID. In this example, for a noise level of $t=1$ the optimal number is $N = 8$, as compared to $N=1$ and $N=16$ (Fig. 4C,D,E). As expected, the ID balances the number of target neurons receiving a signal from the sender neurons with the probability that the signal is transmitted correctly (firing when the sender neurons are firing). On the other hand, the KL measure grows indefinitely with the number of target neurons, with an increasing number of neurons not receiving the correct signal (not firing when the source neurons are firing).

### Comparison to other information measures

The axiomatic characterization of information measures starting from basic properties is an extensive field^1,9–13^. Such measures originally reflected the concerns of a channel designer^1^, and were then extended to an experimenter arbitrating between two hypotheses. In both cases, it is intuitive to design information measures that are additive for all independent probability distributions^9,11,14,15^. The most common properties imposed in information measures are monotonicity, continuity and additivity^10^, which uniquely characterize the KL measure. Note that while the KL measure satisfies our *Causality* property, it does not satisfy *Specificity* or *Intrinsicality*. Symmetry (permutation invariance) is not explicitly required from ID as it is in some axiomatic characterizations of the KL measure^10^, nonetheless, it is a property that both the KL and the ID measures satisfy.

The properties of the KL measure guarantee the existence of an optimal encoding, which defines the channel capacity^16^. In this sense, the KL measure is capturing the average amount of information that can be recovered using error correction codes. Additive measures such as the KL are also widely used in the analysis of complex systems, including neural networks and artificial organisms^2,4^. In this case, the system or its components are analyzed from the perspective of an extrinsic observer viewing the system as an information channel. However, the information that an extrinsic observer may associate with the system does not necessarily reflect the information that elements within the system are actually able to receive or send. This is because the system elements typically lack the ability to control their source or target alphabet and implement error correcting codes. In other words, they cannot choose which symbol was sent or how their symbols will be received. From the extrinsic perspective of a channel designer, a channel has a fixed capacity. KL measures the amount of information that can be potentially transmitted, on average. From the intrinsic perspective, the physical channel has a fixed amount of intrinsic information, rather than a fixed capacity. ID measures the amount of information that is actually transmitted, for a specific input and output.

Here we have argued that, from the intrinsic perspective of a system, information should satisfy expansion (additivity) for independent noiseless probability distributions but dilution (sub-additivity) for independent noisy probability distributions, as required by the intrinsicality property. Sub-additivity for dependent probability distributions is a common requirement in the axiomatic characterization of information measures^9,10,14^, but ID enforces sub-additivity for certain independent probability distributions. Measures with sub-additivity for independent probability distributions have been developed as theoretical generalizations of additive measures^17,18^ as well as for specific applications (e.g., non-conservative systems^19^). However, such measures are sub-additive for all independent probability distributions. By contrast, the ID measure is sub-additive for some independent probability distributions (containing any $p_\alpha < 1$) and additive for others (containing any $p_\alpha = 1$).

Specificity is another characteristic property of intrinsic information, which requires the measure to reflect one specific state—the most informative one—instead of the sum (or generalized mean) over all the states in the probability distribution. By comparison, the Rényi entropy^13^ favours states with high (or low) information within a generalized mean (Kolmogorov–Nagumo mean) depending on a free parameter. In the limit, as the free parameter goes to infinity, this generalized mean puts all the weight on a single state with the highest information, which is useful in cryptography^20^. However, while this generalized mean satisfies specificity, by design it is always additive for all independent probability distributions, and therefore lacks dilution.

## Conclusion

In summary, we have introduced an information measure, the intrinsic difference (ID), that reflects the intrinsic perspective of elements of a system, rather than the extrinsic perspective of a channel designer. From the extrinsic perspective of a designer, the goal is to maximize information transmission, on average, *between* sender and receiver across a given channel. As is well known, this can be done using optimal codes and error-correction strategies. From the intrinsic perspective of system elements, however, the goal is to choose an input and output channel that maximize, at every transmission, the information that is received and sent correctly *from and to* the system. As we show here, this can be done by selecting the scope of inputs and outputs, for example through changes in connection strength, as might be the case with neurons within the cerebral cortex.

Formally, the ID is uniquely characterized by three properties: causality, specificity, and intrinsicality. Notably, the intrinsicality property mandates a balance between expansion and dilution. In this way, optimizing the ID as a measure of intrinsic information allows elements in a network to achieve an optimal trade-off between how much information they could potentially transmit among themselves and how likely they are to actually transmit that information.

The present work motivated and introduced the new measure, proved its uniqueness, and briefly explored how simplified neurons can optimize their inputs and outputs according to intrinsic information. Future work will have to address more realistic elements, the influence of connection patterns, of correlated signals, of signal composition and integration, as well as the effects of learning.

## Supplementary information

Supplementary Information.
