# Supplementary material for: A measure for intrinsic information
Source: Sci Rep. 2020 Nov 2;10:18803. doi: 10.1038/s41598-020-75943-4 (PMC7606539; doi:10.1038/s41598-020-75943-4)
Supplement: Supplementary file 1 — Supplementary Information. [file 41598_2020_75943_MOESM1_ESM.pdf]

Supplementary Material: A Measure for Intrinsic Information

Leonardo S. Barbosa[a], William Marshall[a,b], Sabrina Streipert[a,c], Larissa Albantakis[a], and Giulio Tononi[a,*]

[a]*Department of Psychiatry, University of Wisconsin–Madison, Madison, WI 53719*
[b]*Department of Mathematics and Statistics, Brock University, St. Catharines, ON L2S 3A1*
[c]*Department of Mathematics and Statistics, McMaster University, Hamilton, ON L8S4K1*
[*]*Corresponding author: gtononi@wisc.edu*

1 Supplementary Examples

1.1 Correlated noise

In this work we did not require any specific properties from ID that specify correlated probability distributions. However, one may wonder what is the behavior of the measure when two correlated random variables are considered. Here we inspect the behavior of a channel with two wires where the level of noise in each wire is varied concomitantly from fully noisy ($r = \frac{1}{2}$) to fully constrained ($r = 1$). At the same time, we vary the correlation from independent ($s = 0$) to fully correlated ($s = 1$). The probability in the channel is $P = [p_1, p_2, p_3, p_4]$ where

$$\begin{aligned} p_1 &= a, \\ p_2 = p_3 &= 1 - r - a, \\ p_4 &= a + 2r - 1, \end{aligned}$$

for $a = (1-r)^2 + sr(1-r)$ and $\mathrm{Q} = \left[\frac{1}{4}, \frac{1}{4}, \frac{1}{4}, \frac{1}{4}\right]$. In Supplementary Material Figure S1 we see that for low levels of noise and correlation, ID associates less *ibits* to the channel than the number of bits measured by KL, similar to the results for uncorrelated channels. However, the correlation can shift the amount of noise necessary for ID to associate less *ibits* to the channel than the amount of bits measured by KL when the channel is close to fully noisy.

1.2 Two noiseless bits channel

Although in the main text we start with a channel that has one noiseless wire and is expanded by seven fully noisy wires, this is not a theoretical requirement

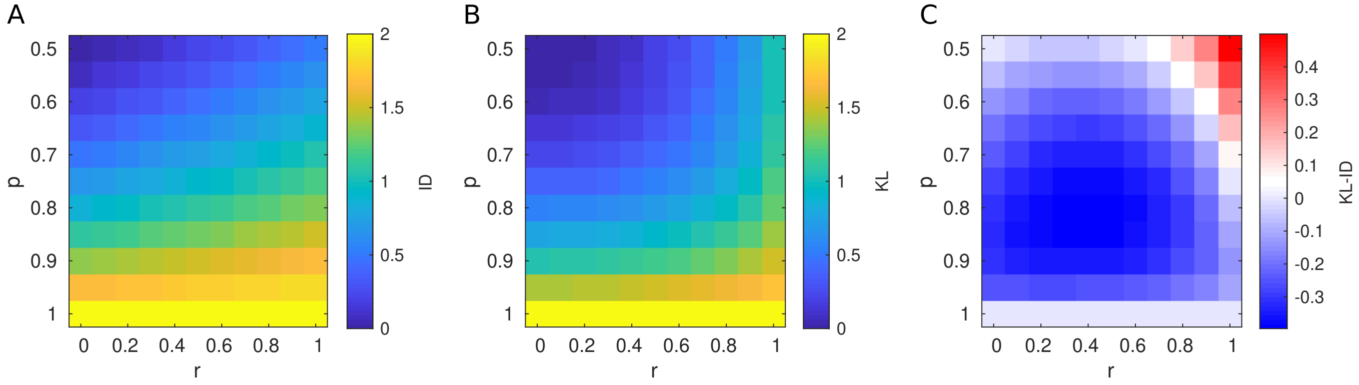

Figure S1: Two noisy wires channel with correlated noise. In the three panels, the x-axis shows the correlation between the two wires and the y-axis shows the probability of each wire correctly transmitting the bit chosen by the sender. A ID values for a channel composed of two such wires; B KL values; C difference between ID and KL. In all panels chancel is the equiprobable distribution.

for all the conclusions in the paper. It is simply an intuitive way to describe the *Intrinsicality* property, composed by expansion and dilution. Indeed, if we start with two noiseless bits and expand until we have six extra fully noisy bits (Supplementary Material Figure S2), a similar conclusion can be reached, although here the probability in the noisy wires composing the channel that has as much intrinsic information as the channel with two noiseless wires is different, i.e., $r \approx 0.82$ instead of $r \approx 0.78$ as in Section 3.2.

1.3 Optimal channel size given maximum accepted error rate

When designing information channels, it is not always possible to implement optimal error correction codes. One common example is when the length of transmissions are restricted, such as real time communications [3]. The common solution to this problem is to establish the best error correction possible given a maximum acceptable error rate [2]. With this additional parameter as a cost function, it is possible to chose the channel size that has the maximum KL with error rate under the maximum accepted value. Here we show how one can use KL to select the optimal channel in the setup used in Section 3.3, where the channel has N equally noise wires, each transmitting the bit selected by the source with probability $s = 0.88$. As we can see in the Supplementary Figure S3, for each maximum accepted error ϵ, one can chose the channel with N wires that has maximum KL and error rate still under ϵ. Notice that ID does not require an extra parameter, and as discussed in Section 3.3, ID will be maximal for $N = 8$.

As a more practical example, imagine that this channel is used to transmit passwords. In general, it is known that a small channel will have a lower error

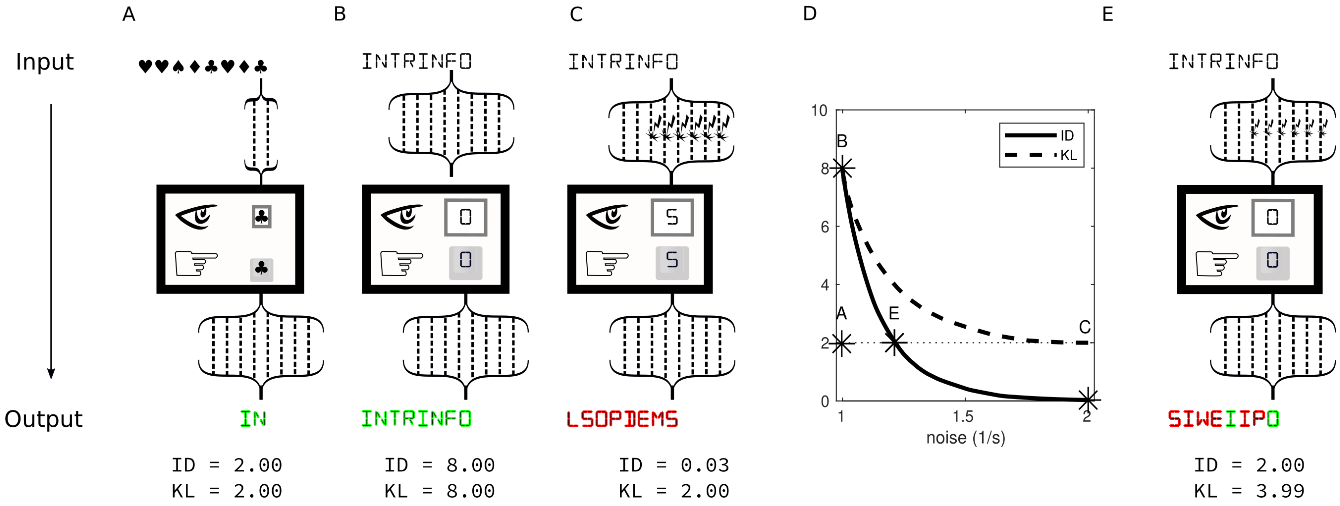

Figure S2: Intrinsic information for two noiseless wires. As in the main text, to facilitate comparison, all output channels are eight-bit noiseless wires that reproduce exactly what was sent. For two-bit-sized input channels, four key presses are accumulated in the encoder to reproduce one output character every eight bits, representing a common read-out. The colors represent whether the output character is correct (green) or incorrect (red). (A) A two-wire input channel that is noiseless, where each suit symbol represents one possible character in the alphabet. The channel transmits the original signal with probability $p = 1$ (noiseless two-bits-channel). (B) An eight-wire input channel with 6 additional wires that are also noiseless and transmit the original signal with probability $p_i = 1, i \in \{1, \ldots, 8\}$ (noiseless byte-channel). (C) An input channel with two noiseless wires and 6 fully noisy wires that transmit the original signal with probability $p_i = 1, i \in \{1, 2\}$ and $p_j = \frac{1}{2}, j \in \{3, \ldots, 8\}$ (noisy byte-channel). Both the intrinsic difference measure (ID) and the KL measures attribute the same amount of information to the noiseless two-bit-channel and the noiseless byte-channel (2 and 8, respectively). However, the KL measure attributes 2 bits to the noisy byte-channel, whereas the ID measure attributes to it close to 0 *ibits*. This is because the ID reflects the amount of information available from the perspective of the receiver in the sealed enclosure (intrinsic information), whereas KL reflects the information available from the extrinsic perspective of a channel designer, which could be extracted using error correcting codes. (D) A byte-channel with additional wires that transmit the original signal with decreasing probability r (or increasing levels of noise $\frac{1}{r}$), from $r = 1$ (noiseless) to $r = \frac{1}{2}$ (fully noisy). Otherwise saying, the probability in the wires are $p_i = 1, i \in \{1, 2\}$ and $p_j = r, j \in \{3, \ldots, 8\}$. Due to the additivity property, the KL measure (dashed line) will always attribute to the byte-channel 2 bit of information or more. By contrast, ID starts from nearly 8 *ibits* and decreases to nearly 0 *ibits*. (E) Due to the smoothness of ID, there is a level of noise ($r \approx 0.82$) for which a byte-channel conveys 2 *ibits*. On a typical run with 4 transmissions such a byte-channel outputs two correct byte-size characters, similar to the noiseless two-bit-channel, exemplifying the balance between expansion and dilution for increasing levels of noise r.

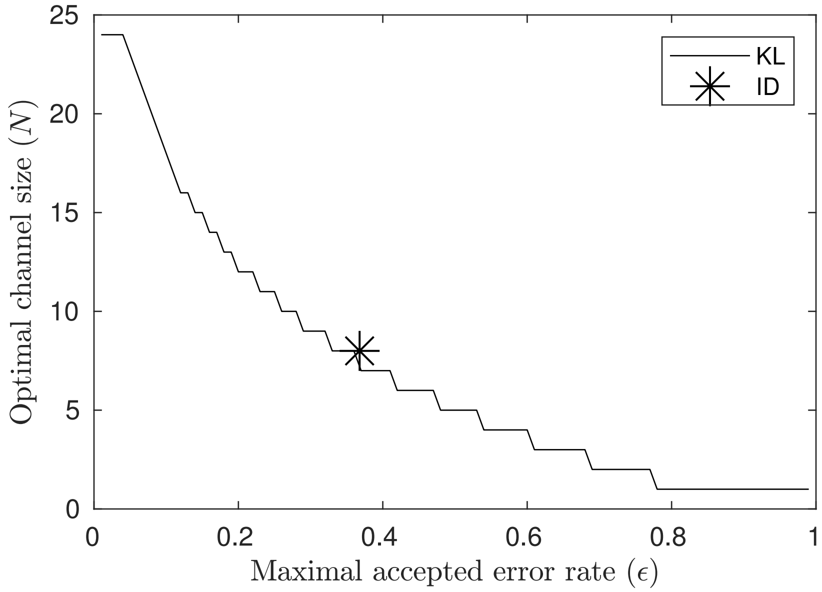

Figure S3: Optimal channel size using the maximum KL with error rate bellow the maximum accepted value. This example considers the same channels used in Section 3.3 of the main text, where the channel has N different wires, each with a probability $s = 0.88$ of correctly transmitting the bit chosen by the sender. The x-axis shows ϵ, that is the maximum error rate accepted by the designer. The y-axis shows N, that is the number of wires in the channel. The solid line defines the optimal channel according to KL, that is the channel with maximum KL that still has error rate bellow ϵ. The black star shows the size of the channel with maximum ID ($N = 8$) as discussed in Section 3.3. The channel with maximum ID will have a principled error rate ($\epsilon \approx 0.37$), which we believe will be useful in many applications.

rate, but it also transmits a password that is simple and subject to brute force exploits. At the same time, a large channel is able to transmit longer passwords that are more secure but at the cost of a higher error rate. Maximizing ID will result in a channel for transmitting passwords that balances reliability and security. While it is possible to establish an ϵ for some particular use of the passwords transmitted in the channel, this particular use may not be known at the time of the design, which forbids the user to define a meaningful maximum acceptable error rate. In this case, we argue that the channel with maximum ID have a principled error rate which transmits the maximum number of correct bits (informativeness) when only considering correct the bits jointly transmitted in one symbol from the sender's alphabet, which may be useful in several applications.

2 Proof

We now give the full statement and proof of the theorem demonstrating the uniqueness of the function defined in Eq. 1 in the main text. We start with

some preliminary definitions:

$$\mathbb{R}^+ = \{x \in \mathbb{R} \colon x > 0\}, \quad \mathbb{N}_2 = \{2, 3, 4, \ldots\},$$

$$\mathrm{J} = (0,1), \quad \hat{\mathrm{J}} = (0,1], \quad \bar{\mathrm{J}} = [0,1], \quad \mathrm{K} = (\bar{\mathrm{J}} \times \bar{\mathrm{J}}) \setminus (\hat{\mathrm{J}} \times \{0\}),$$

$$\mathrm{I}_\delta(x) = \{y \in \mathrm{J} \colon |x - y| < \delta\}.$$

For $n \in \mathbb{N}_2$, we further define

$$\Gamma^n = \left\{ X^n = (x_1, \ldots, x_n) \colon x_1, \ldots, x_n \in \bar{\mathrm{J}}\,; \sum_{\alpha=1}^{n} x_\alpha = 1 \right\},$$

$$V^n = (1, 0, \ldots, 0) \in \Gamma^n, \qquad U^n = \left(\frac{1}{n}, \ldots, \frac{1}{n}\right) \in \Gamma^n,$$

$$\Delta^n = \{(X^n, Y^n) \in \Gamma^n \times \Gamma^n \colon (x_\alpha, y_\alpha) \in \mathrm{K},\ \forall\, \alpha \in \{1, \ldots, n\}\}.$$

Using these definitions, we further define the following properties.

Property I : *Causality.* Let $(P^n, Q^n) \in \Delta^n$. The difference $D(P^n, Q^n)$ is defined as $D \colon \Delta^n \to \mathbb{R}$, such that

$$D(P^n, Q^n) = 0 \iff P^n = Q^n.$$

Property II : *Specificity.* The difference must be state-specific, meaning for $f \colon \mathrm{K} \longrightarrow \mathbb{R}$, $\exists\, \alpha \in \{1, \ldots, n\}$ such that $D(P^n, Q^n) = f(p_\alpha, q_\alpha)$. More precisely, we define

$$D(P^n, Q^n) \coloneqq \max_\alpha \{f(p_\alpha, q_\alpha)\},$$

where f is continuous on K, analytic on $\hat{\mathrm{J}} \times \mathrm{J}$ and $f(0, q_\alpha)$ is analytic on J.[1]

Property III : *Intrinsicality.* Let $(P^l, Q^l) \in \Delta^l$ and $(P^m, Q^m) \in \Delta^m$. Then

a) expansion: $\quad D(V^l * V^m, P^l * Q^m) = D(V^l, P^l) + D(V^m, Q^m),$

b) dilution: $\quad D(P^l * U^m, Q^l * U^m) = \dfrac{D(P^l, Q^l) + D(U^m, U^m)}{m},$

where $P^l * Q^m = (p_1 q_1, \ldots, p_1 q_m, \ldots, p_l q_1, \ldots, p_l q_m) \in \Gamma^{lm}$.

The following lemma allows the analytic extension of real analytic functions.

[1] Note that the maximum operation may admit non-smooth transformations of the function $f(p, q)$, such as $D(P^n, Q^n) = \max_\alpha\{|f(p_\alpha, q_\alpha)|\}$, which provide additional constraints on the maximization. In this particular case with the absolute value, a similar Theorem can be proved showing that $f(p, q) = k' p \log\left(\frac{p}{q}\right)$ is the only function satisfying all three properties, for $k' \neq 0$.

Lemma 1 (See [4, Proposition 1.2.3]). *If f and g are real analytic functions on an open interval $U \in \mathbb{R}$ and if there is a sequence of distinct points $\{x_n\}_n \in U$ with $x_0 = \lim_{n\to\infty} x_n \in U$ such that*

$$f(x_n) = g(x_n),$$

then

$$f(x) = g(x), \qquad \forall\, x \in U.$$

Corollary 1 (See [4, Corollary 1.2.6]). *If f and g are analytic functions on an open interval U and if there is an open interval $W \subseteq U$ such that*

$$f(x) = g(x) \qquad \forall\; x \in W,$$

then

$$f(x) = g(x) \qquad \forall\; x \in U.$$

The following lemma shows that a strict maximum over continuous functions, each evaluated at fixed points, must hold for an open interval around such fixed points.

Lemma 2. *Let $g_\alpha \colon J \to \mathbb{R}$ be continuous functions, where $\alpha \in \{1, \ldots, n\}$, fix $x_1, \ldots, x_n \in J$. If there exists α^* such that*

$$\max_{\alpha}\{g_\alpha(x_\alpha)\} = g_{\alpha^*}(x_{\alpha^*}),$$

where $g_{\alpha^*}(x_{\alpha^*})$ is a strict maximum, then there exists $\delta > 0$ such that

$$\max_{\alpha}\{g_\alpha(\hat{x}_\alpha)\} = g_{\alpha^*}(\hat{x}_{\alpha^*}),$$

for $\hat{x}_\alpha \in I_\delta(x_\alpha)$ and for all $\alpha \in \{1, \ldots, n\}$.

Proof. Let $\beta \in \{1, ..., n\} \setminus \{\alpha^*\}$, then

$$g_{\alpha^*}(x_{\alpha^*}) > g_\beta(x_\beta),$$

and there exists $\epsilon_\beta > 0$ such that

$$g_{\alpha^*}(x_{\alpha^*}) - g_\beta(x_\beta) = \epsilon_\beta.$$

Let $d \in$ J such that $x_{\alpha^*} + d, x_\beta - d \in$ J and $e \coloneqq g_{\alpha^*}(x_{\alpha^*} + d) - g_\beta(x_\beta - d)$. Then

$$\begin{aligned} &g_{\alpha^*}(x_{\alpha^*}) - g_\beta(x_\beta) = \epsilon_\beta - e + e, \\ &e = \epsilon_\beta - [g_{\alpha^*}(x_{\alpha^*}) - g_{\alpha^*}(x_{\alpha^*} + d)] - [g_\beta(x_\beta - d) - g_\beta(x_\beta)]. \end{aligned} \tag{1}$$

By the continuity of g_{α^*} and g_β, for $\epsilon'_\beta = \frac{\epsilon_\beta}{2} > 0$, there exists $\delta_\beta > 0$ such that

$$\left.\begin{aligned} &|g_{\alpha^*}(x_{\alpha^*}) - g_{\alpha^*}(x_{\alpha^*} + d)| &< \epsilon'_\beta \\ &|g_\beta(x_\beta - d) - g_\beta(x_\beta)| &< \epsilon'_\beta \end{aligned}\right\} \qquad \text{for} \quad |d| < \delta_\beta.$$

By Eq. (1) we have

$$e > \epsilon_\beta - \frac{\epsilon_\beta}{2} - \frac{\epsilon_\beta}{2} = 0,$$

which implies, by definition of e,

$$g_{\alpha^*}(x_{\alpha^*} + d) - g_\beta(x_\beta - d) > 0,$$

i.e.

$$g_{\alpha^*}(x_{\alpha^*} + d) > g_\beta(x_\beta - d).$$

Since this holds for all $\beta \in \{1, ..., n\} \setminus \{\alpha^*\}$, it suffices to require that

$$\delta = \min_{\beta \in \{1,...,n\} \setminus \{\alpha^*\}} \{\delta_\beta\},$$

which completes the proof. □

We now provide the solution to a functional equation similar to the Pexider logarithmic equation ([1]).

Lemma 3. *Let $f, g, h\colon J \to \mathbb{R}$ be analytic functions on J. If the functional equation*

$$f(pq) = \max\{g(p), h(p)\} + \max\{g(q), h(q)\},$$

holds for $pq \in I_\delta(p'q')$ where $I_\delta(p'q') \subseteq J$, then

$$f(x) = c\log(x) + d, \qquad c, d \in \mathbb{R},\ x \in J.$$

Proof. First suppose that there exists $(p_i, q_i) \in \mathrm{J} \times \mathrm{J}$ such that $p_i q_i \in I_\delta(p'q')$ and for some $i \in \{g, h\}$

$$i(p_i) = \max\{g(p_i), h(p_i)\}$$

is a strict maximum. Then, by Lemma 2, there exists $\delta_p > 0$ such that

$$i(p) = \max\{g(p), h(p)\}, \qquad p \in \mathrm{I}_{\delta_p}(p_i).$$

Second if there does not exist $(p_i, q_i) \in \mathrm{J} \times \mathrm{J}$ such that $p_i q_i \in I_\delta(p'q')$ and $i(p_i)$ is a strict maximum, then we set $q_i = q'$, $p_i = p'$ and $\delta_p = \frac{\delta}{q'}$ so that Eq. (2) holds since $g(p) = h(p)$ for all $(p, q) \in \mathrm{J} \times \mathrm{J}$ such that $pq \in \mathrm{I}_\delta(p'q')$. Next define $\delta' := \min\{\delta - |p_i q_i - p'q'|, \delta_p q'\}$ and suppose that there exists $q_j \in \mathrm{J}$ such that $p_i q_j \in I_{\delta'}(p_i q_i)$ and that for some $j \in \{g, h\}$

$$j(q_j) = \max\{g(q_j), h(q_j)\}$$

is a strict maximum. Then, by Lemma 2, there exists $\delta_q > 0$ such that

$$j(q) = \max\{g(q), h(q)\}, \qquad q \in \mathrm{I}_{\delta_q}(q_j).$$

Finally, if there does not exist $q_j \in \mathrm{J}$ such that $p_i q_j \in I_{\delta'}(p_i q_i)$ and $j(q_j)$ is a strict maximum, then we set $q_j = q_i$ and $\delta_q = \frac{\delta'}{p_i}$ so that Eq. (2) holds since $g(q) = h(q)$ for all $(p, q) \in \mathrm{J} \times \mathrm{J}$ such that $pq \in \mathrm{I}_{\delta'}(p_i q_i)$. Let $pq = x$ and define $\delta'' := \min\{\delta' - |p_i q_j - p_i q_i|, \delta_q p_i\}$, then

$$f(x) = i(p) + j(q), \qquad x \in \mathrm{I}_{\delta''}(p_i q_j).$$

Since the functions are analytic on J and therefore twice differentiable, then

$$\frac{\partial}{\partial q}\left[\frac{\partial}{\partial p}[f(x)]\right] = x\frac{d}{dx^2}f(x) + \frac{d}{dx}f(x) = \frac{\partial}{\partial q}\left[\frac{\partial}{\partial p}i(p)\right] + \frac{\partial}{\partial q}\left[\frac{\partial}{\partial p}j(q)\right] = 0.$$

Integrating with respect to x yields

$$f(x) = c\log(x) + d,$$

for $c, d \in \mathbb{R}$ and $x \in \mathrm{I}_{\delta'}(x')$ where $x' = p'q'$. Since f is analytic on J and since $\mathrm{I}_{\delta'}(x') \subset \mathrm{J}$, by Corollary 1, we can extend $f(x)$ such that

$$f(x) = c\log(x) + d,$$

for $c, d \in \mathbb{R}$ and $x \in \mathrm{J}$. □

Lemma 4. *Let $(P^n, P^n) \in \Delta^n$ for $n \in \mathbb{N}_2$. If $D\colon \Delta^n \to \mathbb{R}$ satisfies Properties I and II then*

$$f(p, p) = 0.$$

Proof. Let $P^2 = (p, 1-p)$ for $p \in \bar{\mathrm{J}}$ and define $F(p) := f(p, p)$. By Properties I and II, we have

$$D(P^2, P^2) = \max\{F(p), F(1-p)\} = 0.$$

For $p = \frac{1}{2}$,

$$\max\left\{F\left(\frac{1}{2}\right), F\left(\frac{1}{2}\right)\right\} = F\left(\frac{1}{2}\right) = 0.$$

By Property I, Eq. (2) must hold for all $p \in \mathrm{J}$ and since $F(p)$ is continuous and analytic in J by Property II', F is smooth and therefore,

$$F'(p) = 0, \qquad \forall\, p \in \mathrm{J},$$

is the only solution that does not violate Property I. To agree, note first that if $F\left(\frac{1}{2}\right)$ is a maximum then $F'\left(\frac{1}{2}\right) = 0$ so that there exists $\delta > 0$ such that $F(\frac{1}{2} + \delta) < 0$ and $F(\frac{1}{2} - \delta) < 0$. Hence,

$$\max\left\{F\left(\frac{1}{2} + \delta\right), F\left(\frac{1}{2} - \delta\right)\right\} < 0,$$

which violates Eq. (2). Second, if $F\left(\frac{1}{2}\right)$ is not a maximum and Eq. (2) does not hold, then there exists $\delta > 0$ such that at least one of the following must be true

$$\left[F'\left(\frac{1}{2}\right) > 0\right] \vee \left[\left(F'\left(\frac{1}{2}\right) = 0\right) \wedge \left(F'\left(\frac{1}{2}+\delta\right) > 0\right)\right] \implies F\left(\frac{1}{2}+\delta\right) > 0,$$

$$\left[F'\left(\frac{1}{2}\right) < 0\right] \vee \left[\left(F'\left(\frac{1}{2}\right) = 0\right) \wedge \left(F'\left(\frac{1}{2}-\delta\right) < 0\right)\right] \implies F\left(\frac{1}{2}-\delta\right) > 0,$$

by which

$$\max\left\{F\left(\frac{1}{2}+\delta\right), F\left(\frac{1}{2}-\delta\right)\right\} > 0.$$

This also violates Eq. (2) and therefore Eq. (2) is the only solution which does not violate Property I. This implies that $F(p) = f(p,p) = c$ is a constant where $f\left(\frac{1}{2},\frac{1}{2}\right) = c = 0$. By Property II', f is continuous and therefore $f(p,p) = 0$ for all $p \in \bar{\mathrm{J}}$. □

We now prove our main result.

Theorem 1. *Let* $(P^n, Q^n) \in \Delta^n$ *for* $n \in \mathbb{N}_2$ *and* $D\colon \Delta^n \to \mathbb{R}$ *where* D *satisfies Properties I, II and III. Then*

$$D(P^n, Q^n) = \max_{\alpha}\{f(p_\alpha, q_\alpha)\},$$

where

$$f(p,q) = k\,p\log\left(\frac{p}{q}\right), \qquad (p,q) \in K,\ k \in \mathbb{R}^+. \tag{2}$$

Proof of Theorem 1. First we show that the function in Eq. (2) satisfies Properties I, II and III. To see that the function satisfies Property I, notice that for $(P^n, Q^n) \in \Delta^n$ where $n \in \mathbb{N}_2$ and $P^n \neq Q^n$, there exists $\beta \in \{1,\ldots,n\}$ such that

$$p_\beta > q_\beta \implies \log\left(\frac{p_\beta}{q_\beta}\right) > 0.$$

At the same time, since $k > 0$, we have

$$kp_\beta\log\left(\frac{p_\beta}{q_\beta}\right) > 0 \implies D(P^n, Q^n) = \max_\alpha\left\{kp_\alpha\log\left(\frac{p_\alpha}{q_\alpha}\right)\right\} \geq p_\beta\log\left(\frac{p_\beta}{q_\beta}\right) > 0,$$

while for $P^n = Q^n$, we have

$$D(P^n, P^n) = \max_\alpha\left\{kp_\alpha\log\left(\frac{p_\alpha}{p_\alpha}\right)\right\} = 0.$$

It is clear that the function f in Eq. (2) satisfies Property II. To see that it satisfies Property III.a, for $(V^l, P^l) \in \Delta^l$ and $(V^m, Q^m) \in \Delta^m$, note that

$$\begin{aligned} D(V^l * V^m, P^l * Q^m) &= \max\left\{k \log\left(\frac{1}{p_1 q_1}\right), 0\right\} \\ &= k \log\left(\frac{1}{p_1 q_1}\right) = k\log\left(\frac{1}{p_1}\right) + k \log\left(\frac{1}{q_1}\right) \\ &= \max\left\{k \log\left(\frac{1}{p_1}\right), 0\right\} + \max\left\{k \log\left(\frac{1}{q_1}\right), 0\right\} \\ &= D(V^l, P^l) + D(V^m, Q^m). \end{aligned}$$

Similarly by Property III.b, notice that for $(P^l, Q^l) \in \Delta^l$, we have

$$\begin{aligned} D(P^l * U^m, Q^l * U^m) &= \max_\alpha \left\{\frac{kp_\alpha}{m} \log\left(\frac{p_\alpha}{q_\alpha}\right)\right\} = \frac{1}{m} \max_\alpha \left\{kp_\alpha \log\left(\frac{p_\alpha}{q_\alpha}\right)\right\} \\ &= \frac{1}{m} D(P^l, Q^l). \end{aligned}$$

The remaining part of the proof is divided into two steps:

Step 1. First we show that under four assumptions, f satisfies Properties I, II and III if, and only if, $f(p,q) = kp \log\left(\frac{p}{q}\right)$ for $k \in \mathbb{R}^+$.

Step 2. Next we show that if any of our assumptions is violated, then no suitable f exists.

Verification of Step 1. We apply Property III.a with $P^2 = (p, 1-p)$ and $Q^2 = (q, 1-q)$ for some $p, q \in \mathrm{J}$ where $p \neq q$. We then have

$$D(V^2 * V^2, P^2 * Q^2) = D(V^2, P^2) + D(V^2, Q^2).$$

By Property II, the following identity holds

$$\begin{aligned} &\max\{f(1, pq), f(0, p(1-q)), f(0, (1-p)q), f(0, (1-p)(1-q))\} \\ &\quad = \max\{f(1,p), f(0, 1-p)\} + \max\{f(1,q), f(0,1-q)\}. \end{aligned} \tag{3}$$

Our first assumption (A1) states that there exists $p', q' \in \mathrm{J}$ such that $f(1, p'q')$ is a strict maximum. By Lemma 2, there exists $\delta > 0$ such that

$$f(1, pq) = \max\{f(1,p), f(0,1-p)\} + \max\{f(1,q), f(0,1-q)\}, \tag{4}$$

for $pq \in \mathrm{I}_\delta(p'q')$. Further, by Lemma 3

$$f(1,q) = c\log(q) + d, \qquad c, d \in \mathbb{R}, \ \forall\, q \in \mathrm{J},$$

and since by Property II f is continuous, the application of Lemma 4 yields

$$\lim_{q \to 1} f(1,q) = \lim_{q\to 1} c\log(q) + d = d = 0,$$

i.e. for $k = -c$, we have

$$f(1,q) = k \log\left(\frac{1}{q}\right), \qquad \forall\, q \in \mathrm{J}. \tag{5}$$

Now applying Property III.b for $l = 2$, $P^2 = V^2$, $Q^2 = (r, 1-r)$ for $r \in \mathrm{J}$, and $m \in \mathbb{N}_2$, we have

$$D(V^2 * U^m, Q^2 * U^m) = \frac{1}{m} D(V^2, Q^2),$$

and by Property II

$$\max\left\{f\left(\frac{1}{m}, \frac{r}{m}\right), f\left(0, \frac{1-r}{m}\right)\right\} = \frac{1}{m}\max\{f(1,r), f(0,1-r)\}$$

for $r \in \mathrm{J}$. Our second assumption (A2) states that there exists $q_1 \in \mathrm{J}$ such that $f(1,q_1) > f(0, 1-q_1)$. Further, by Lemma 2 and Eq. 5, there exists $\delta > 0$ such that

$$\max\left\{f\left(\frac{1}{m}, \frac{r}{m}\right), f\left(0, \frac{1-r}{m}\right)\right\} = \frac{k}{m}\log\left(\frac{1}{r}\right), \qquad r \in \mathrm{I}_\delta(q_1). \tag{6}$$

Note that (A2) also implies that $k > 0$. To agree, let $q \in \mathrm{J}$ and $Q^2 = (q, 1-q)$. By Properties I and II, we have

$$D(V^2 * V^2, Q^2 * V^2) = \max\{f(1,q), f(0,1-q), f(0,0)\} \neq 0.$$

By Eq. (5) and Lemma 4

$$\max\left\{k\log\left(\frac{1}{q}\right), f(0,1-q), 0\right\} \neq 0, \qquad \forall\, q \in \mathrm{J}.$$

If $k \leq 0$, then the previous equation implies that $f(0, 1-q)$ must be positive, i.e.

$$f(0,1-q) > 0 \geq k\log\left(\frac{1}{q}\right), \qquad \forall\, q \in \mathrm{J},$$

which contradicts our assumption (A2) that there exists $q_1 \in \mathrm{J}$ such that

$$f(1,q_1) = k\log\left(\frac{1}{q_1}\right) > f(0, 1-q_1).$$

Our third assumption (A3) states that $f\left(0, \frac{1-r}{m}\right)$ is a strict maximum in Eq. (6) for a finite number of $m \in \mathbb{N}_2$. More specifically, define

$$\mathcal{M} := \left\{m \in \mathbb{N}_2 \colon\ \exists\, r \in \mathrm{I}_\delta(q_1)\, \text{s.t.}\, f\left(0, \frac{1-r}{m}\right) > f\left(\frac{1}{m}, \frac{r}{m}\right)\right\}.$$

Then (A3) states that $\sup\{\mathcal{M}\} < \infty$ (where $\sup\{\mathcal{M}\} := -\infty$ if $\mathcal{M} = \emptyset$). Define $m_0 := 1$ if $\mathcal{M} = \emptyset$ and $m_0 := \sup\{\mathcal{M}\}$ otherwise. For a fixed $m^* > m_0$, let $q = \frac{r}{m^*}$. Then, by Eq. (6),

$$f\left(\frac{1}{m^*}, q\right) = \frac{k}{m^*}\log\left(\frac{1}{m^* q}\right), \qquad q \in \mathrm{I}_{\frac{\delta}{m^*}}\left(\frac{q_1}{m^*}\right).$$

By Corollary 1, we can extend $f\left(\frac{1}{m^*}, q\right)$ to J, to get

$$f\left(\frac{1}{m^*}, q\right) = \frac{k}{m^*}\log\left(\frac{1}{m^* q}\right), \qquad q \in \mathrm{J}.$$

Since this holds for all $m^* > m_0$, we have for the sequence $\mathrm{h}_0 := \left\{\frac{1}{m+m_0}\right\}_m$

$$f(p,q) = kp\log\left(\frac{p}{q}\right), \qquad p \in \mathrm{h}_0,\ q \in \mathrm{J}. \tag{7}$$

Let $n \in \mathbb{N}_2$ and let $0 < q_2 < \frac{n-1}{2n}$, then $q_2 \in \mathrm{J}$. Applying Property III.b to $l = 2$, $P^2 = \left(\frac{n-1}{2n}, \frac{n+1}{2n}\right)$, $Q^2 = (q_2, 1-q_2)$ and $m = (n-1)(n+1)$, we have

$$D(P^2 * U^m, Q^2 * U^m) = \frac{1}{m} D(P^2, Q^2),$$

and by Property II

$$\max\left\{f\left(\frac{1}{2n(n+1)}, \frac{q_2}{(n-1)(n+1)}\right), f\left(\frac{1}{2n(n-1)}, \frac{1-q_2}{(n-1)(n+1)}\right)\right\} = \frac{1}{(n-1)(n+1)}\max\left\{f\left(\frac{n-1}{2n}, q_2\right), f\left(\frac{n+1}{2n}, 1-q_2\right)\right\}.$$

By Eq. (7), for $2n(n-1) > m_0$, we have

$$\max\left\{\frac{k}{2n(n+1)}\log\left(\frac{n-1}{2nq_2}\right), \frac{k}{2n(n-1)}\log\left(\frac{n+1}{2n(1-q_2)}\right)\right\} = \frac{1}{(n-1)(n+1)}\max\left\{f\left(\frac{n-1}{2n}, q_2\right), f\left(\frac{n+1}{2n}, 1-q_2\right)\right\},$$

and since $k > 0$ and $q_2 < \frac{n-1}{2n} < \frac{1}{2}$, this yields

$$\frac{k(n-1)}{2n}\log\left(\frac{n-1}{2nq_2}\right) = \max\left\{f\left(\frac{n-1}{2n}, q_2\right), f\left(\frac{n+1}{2n}, 1-q_2\right)\right\}.$$

Define $n_0 := \inf\{n \in \mathbb{N}_2 \colon 2n(n-1) > m_0\}$. Then, for the sequence $\mathrm{h}_{\frac{1}{2}} := \left\{\frac{n+n_0-1}{2(n+n_0)}\right\}_n$, we have

$$kp_n\log\left(\frac{p_n}{q}\right) = \max\left\{f\left(p_n, q\right), f\left(1-p_n, 1-q\right)\right\}, \tag{8}$$

for $(p_n, q) \in \mathrm{h}_{\frac{1}{2}} \times (0, p_n)$.

Our fourth and last assumption, (A4), is that $f(1-p_n, 1-q)$ is a strict maximum only for a finite number of $p_n \in \mathrm{h}_{\frac{1}{2}}$. More specifically, let

$$\mathcal{P} = \{p_n \in \mathrm{h}_{\frac{1}{2}} : \ \exists\, q \in (0, p_n) \ \text{such that} \ f(1-p_n, 1-q) > f(p_n, q)\}.$$

Then (A4) states that $\sup\{\mathcal{P}\} < \frac{1}{2}$ where, for convention, $\sup\{\mathcal{P}\} := -\infty$ if $\mathcal{P} = \emptyset$. Define $n' := n_0$ if $\mathcal{P} = \emptyset$, else there exists $n' \in \mathbb{N}_2$ such that $\frac{n'-1}{2n'} = \sup\{\mathcal{P}\}$. Define $\mathrm{h}'_{\frac{1}{2}} := \{\frac{n+n'-1}{2(n+n')}\}_n$, then

$$f\left(p_n, q\right) = k p_n \log\left(\frac{p_n}{q}\right), \qquad (p_n, q) \in \mathrm{h}'_{\frac{1}{2}} \times (0, p_n). \tag{9}$$

By Corollary 1, for a fixed $p_n^* \in \mathrm{h}'_{\frac{1}{2}}$, we can extend $f(p_n^*, q)$ to J, i.e.

$$f(p_n^*, q) = k p_n^* \log\left(\frac{p_n^*}{q}\right), \qquad q \in \mathrm{J}.$$

Finally, for a fixed $q^* \in \mathrm{J}$, by Lemma 1, we can uniquely extend $f(p_n, q^*)$ to J such that

$$f(p, q^*) = k p \log\left(\frac{p}{q^*}\right), \qquad p \in \mathrm{J}.$$

Since this holds for all $q^* \in \mathrm{J}$, we have

$$f(p, q) = k p \log\left(\frac{p}{q}\right), \qquad (p, q) \in (\mathrm{J} \times \mathrm{J}). \tag{10}$$

By Property II, f is continuous in K and the following limits exist for all $p, q \in \mathrm{J}$:

$$\begin{aligned}
f(1, q) &= \lim_{p \to 1^-} k p \log\left(\frac{p}{q}\right) = k \log\left(\frac{1}{q}\right), \\
f(p, 1) &= \lim_{q \to 1^-} k p \log\left(\frac{p}{q}\right) = k p \log(p), \\
f(0, q) &= \lim_{p \to 0^+} k p \log\left(\frac{p}{q}\right) = 0, \\
f(0, 1) &= \lim_{p \to 0^+} k p \log(p) = 0, \\
f(0, 0) &= \lim_{p \to 0^+} k p \log\left(\frac{p}{p}\right) = 0, \\
f(1, 1) &= \lim_{p \to 1^-} k p \log\left(\frac{p}{p}\right) = 0.
\end{aligned}$$

Consequently, f is also the only function which satisfies Property III.b for $l = m = 2$ and $(p, q) \in \mathrm{K}$, i.e.

$$f(p,q) = kp \log\left(\frac{p}{q}\right), \qquad (p,q) \in \mathrm{K},\ k \in \mathbb{R}^+. \tag{2}$$

Verification of Step 2. Up until here we have showed that Eq. (2) not only defines a function which satisfies Properties I, II and III, but it also defines the *only* function which satisfies Properties I, II and III for $l = m = 2$ given the following assumptions

A1: $\exists\, p', q' \in \mathrm{J}$ such that $f(1, p'q')$ is a strict maximum on Eq. (2),

A2: $\exists\, q_1 \in \mathrm{J}$ such that $f(1, q_1) > f(0, 1 - q_1)$,

A3: $\sup\{\mathcal{M}\} < \infty$,

A4: $\sup\{\mathcal{P}\} < \frac{1}{2}$.

All that is left to prove the theorem is to show that violating any of these assumptions also violates some property. First assume that (A1), (A2) and (A3) are true but (A4) is violated, i.e. $\sup\{\mathcal{P}\} = \lim_{n\to\infty} p_n = \frac{1}{2}$ for $p_n \in \mathrm{h}_{\frac{1}{2}}$. Let $p'_n = 1 - p_n$, $q' = 1 - q$ and let $\mathrm{g} = \{1 - p_i\}_i$ be the sequence of ordered elements in $\mathcal{P}$ such that $p_i < p_{i+1}$. Then by Eq. (8), for all $p'_n \in \mathrm{g}$, there exists $q' \in (p'_n, 1)$ such that

$$f(p'_n, q') = k(1 - p'_n) \log\left(\frac{1 - p'_n}{1 - q'}\right). \tag{11}$$

By Lemma 2, for a fixed $p^*_n \in \mathrm{g}$, there exists $\delta' > 0$ such that

$$f\left(p^*_n, q\right) = k(1 - p^*_n) \log\left(\frac{1 - p^*_n}{1 - q}\right), \qquad q \in \mathrm{I}_{\delta'}(q').$$

Since this holds for all $p^*_n \in \mathrm{g}$, we have

$$f\left(p'_n, q\right) = k(1 - p'_n) \log\left(\frac{1 - p'_n}{1 - q}\right), \qquad (p'_n, q) \in \mathrm{g} \times \mathrm{I}_{\delta'}(q').$$

Similarly to Eq. (9) this result can be extended to $\mathrm{J} \times \mathrm{J}$, i.e.

$$f(p,q) = k(1 - p) \log\left(\frac{1 - p}{1 - q}\right), \qquad (p,q) \in (\mathrm{J} \times \mathrm{J}). \tag{12}$$

Applying Property III.b to $l = m = 2$, $P^2 = (p, 1 - p)$, $Q^2 = (q, 1 - q)$ with $q \neq p$ yields

$$D(P^2 * U^2, Q^2 * U^2) = \frac{1}{2} D(P^2, Q^2).$$

Further, by Property II

$$\max\left\{f\left(\frac{p}{2},\frac{q}{2}\right),f\left(\frac{1-p}{2},\frac{1-q}{2}\right)\right\}=\frac{1}{2}\max\left\{f(p,q),f(1-p,1-q)\right\}.$$

However this contradicts Eq. (12) since

$$\max\left\{k\left(1-\frac{p}{2}\right)\log\left(\frac{1-\frac{p}{2}}{1-\frac{q}{2}}\right),k\left(1-\frac{1-p}{2}\right)\log\left(\frac{1-\frac{1-p}{2}}{1-\frac{1-q}{2}}\right)\right\}$$
$$\neq\frac{1}{2}\max\left\{k\left(1-p\right)\log\left(\frac{1-p}{1-q}\right),kp\log\left(\frac{p}{q}\right)\right\}.$$

Next we assume that (A1) and (A2) are true but (A3) is violated, meaning that $\sup\{\mathcal{M}\}=\infty$. In this case, for all $m'\in\mathbb{N}_2$, there exists $(m',r')\in\mathbb{N}_2\times\mathrm{I}_\delta(q_1)$ such that $f\left(0,\frac{1-r}{m}\right)$ is a strict maximum in Eq. (6), i.e.

$$f\left(0,\frac{1-r'}{m'}\right)=\frac{k}{m'}\log\left(\frac{1}{r'}\right). \tag{13}$$

For $q'=\frac{1-r'}{m'}\in\mathrm{I}_{\frac{\delta}{m'}}\left(\frac{1-q_1}{m'}\right)$

$$f\left(0,q'\right)=\frac{k}{m'}\log\left(\frac{1}{1-m'q'}\right).$$

By Lemma 2, there exists $\delta'>0$ such that

$$f\left(0,q\right)=\frac{k}{m'}\log\left(\frac{1}{1-m'q}\right),\qquad q\in\mathrm{I}_{\delta'}\left(q'\right).$$

By Lemma 1, we can extend $f\left(0,q\right)$ to $\left(0,\frac{1}{m'}\right)$, i.e.

$$f\left(0,q\right)=\frac{k}{m'}\log\left(\frac{1}{1-m'q}\right),\qquad q\in\left(0,\frac{1}{m'}\right).$$

However this implies that f is discontinuous and violates Property II since

$$\lim_{q\to\frac{1}{m'}}f\left(0,q\right)=\lim_{q\to\frac{1}{m'}}\frac{k}{m'}\log\left(\frac{1}{1-m'q}\right)=\pm\infty.$$

We now assume that (A1) is true but (A2) is violated, i.e.

$$f(1,q)\leq f(0,1-q),\qquad\forall\,q\in\mathrm{J}.$$

For $p',q'\in\mathrm{J}$ and $\delta>0$, by Eq. (4) and by Eq. (5)

$$k\log\left(\frac{1}{pq}\right)=f(0,1-p)+f(0,1-q),\qquad pq\in\mathrm{I}_\delta(p'q'). \tag{14}$$

Let $q_1, q_2 \in \mathrm{J}$ such that $p'q_1, p'q_2 \in \mathrm{I}_{\delta'}(p'q')$, then

$$F(q_1) = k\log\left(\frac{1}{q_1}\right) - f(0, 1-q_1) = f(0, 1-p') - k\log\left(\frac{1}{p'}\right)$$
$$= k\log\left(\frac{1}{q_2}\right) - f(0, 1-q_2) = F(q_2).$$

Therefore $F(q) = d$ is constant and

$$f(0, 1-q) = k\log\left(\frac{1}{q}\right) + d, \qquad q \in \mathrm{I}_{\frac{\delta}{p'}}(q').$$

Plugging this back into Eq. (14) we see that $d = 0$ and together with Lemma 1, we have

$$f(0, q) = k\log\left(\frac{1}{1-q}\right), \qquad \forall\, q \in \mathrm{J}.$$

However this is a contradiction since by Eq. (5) for $k \neq 0$ and $x = -\frac{1}{2}\mathrm{sign}(k)$,

$$f(1, x) = k\log\left(\frac{1}{x}\right) > k\log\left(\frac{1}{1-x}\right) = f(0, 1-x).$$

Note that $k = 0$ violates Property I since for any $q \in \mathrm{J}$ and $Q^2 = (q, 1-q) \neq V^2$

$$D(V^2, Q^2) = \max\left\{k\log\left(\frac{1}{q}\right), k\log\left(\frac{1}{1-q}\right)\right\} = 0.$$

Finally if (A1) is violated then there does not exist $p', q' \in \mathrm{J}$ such that $f(1, p'q')$ is a strict maximum on Eq. (2). Hence, for all $p, q \in \mathrm{J}$, there exists $x' \in \{p(1-q), q(1-p), (1-p)(1-q)\}$ such that

$$f(0, x') = \max\{f(1, p), f(0, 1-p)\} + \max\{f(1, q), f(0, 1-q)\}.$$

If $f(0, x')$ is a strict maximum, then by Lemma 2, there exists $\delta > 0$ such that for $\mathrm{I} = \mathrm{I}_\delta(x')$

$$f(0, x) = \max\{f(1, p), f(0, 1-p)\} + \max\{f(1, q), f(0, 1-q)\}, \qquad x \in \mathrm{I}. \quad (15)$$

If there does not exist $x' \in \{p(1-q), q(1-p), (1-p)(1-q)\}$ such that $f(0, x')$ is a strict maximum, then there must exist $x \in \{p(1-q), q(1-p), (1-p)(1-q)\}$ such that Eq. 15 holds for $\mathrm{I} = \mathrm{J}$. In both cases, by Lemma 3

$$f(0, x) = c\log(x) + d, \qquad c, d \in \mathbb{R},\ x \in \mathrm{J}.$$

However this implies that f is discontinuous since

$$\lim_{x\to 0} f(0, x) = \lim_{x\to 0} c\log(x) + d = \pm\infty,$$

even though $f(0, 0) = 0$ by Lemma 4. □

References

[1] J. Aczél. *Lectures on Functional Equations and Their Applications.* Dover Publications, Mineola, N.Y, February 2006.

[2] R. A. Costa, M. Langberg, and J. Barros. One-shot capacity of discrete channels. In *2010 IEEE International Symposium on Information Theory*, pages 211–215, June 2010.

[3] Giuseppe Durisi, Tobias Koch, and Petar Popovski. Toward Massive, Ultrareliable, and Low-Latency Wireless Communication With Short Packets. *Proceedings of the IEEE*, 104(9):1711–1726, September 2016.

[4] Steven G. Krantz and Harold R. Parks. *A Primer of Real Analytic Functions.* Birkhäuser Advanced Texts Basler Lehrbücher. Birkhäuser Basel, 2 edition, 2002.
